# Gender-Affirming Voice Training Delivered by a Novel Mobile App: Mixed Methods Study

**DOI:** 10.2196/96102

**Published:** 2026-10-08

**Authors:** Isaac L Alter, Christopher P Kruglik, Sara W Albert, Rachel Coleman, Mary Pitti, Alexandra Li, Katerina Andreadis, David Bayne, Eseosa Odigie, Iyra Chandra, Valentina Mocchetti, David Liao, Keith A Chadwick, Anaïs Rameau

**Affiliations:** 1 Weill Cornell Medical College, Department of Otolaryngology‐Head and Neck Surgery, Sean Parker Institute for the Voice, 240 E 59th St, New York, NY, United States, 1 646-962-7464; 2 Robert Larner, M.D. College of Medicine at the University of Vermont, Burlington, VT, United States; 3 Washington University School of Medicine, St. Louis, MO, United States; 4 Department of Speech-Language Pathology and Audiology, Ithaca College, Ithaca, NY, United States; 5 Department of Population Health Sciences, New York University Grossman School of Medicine, New York, NY, United States; 6 Department of Otolaryngology-Head and Neck Surgery, Duke University School of Medicine, Durham, NC, United States; 7 Wake Forest University School of Medicine, Winston-Salem, NC, United States; 8 Department of Otolaryngology-Head and Neck Surgery, University of Pennsylvania Perelman School of Medicine, Philadephia, PA, United States; 9 Department of Surgery, Division of Otolaryngology-Head & Neck Surgery, Stony Brook University, Stony Brook, NY, United States

**Keywords:** transgender persons, gender-affirming, voice training, mobile apps, qualitative research, mixed methods

## Abstract

**Background:**

Gender-affirming voice training (GAVT) has reduced gender incongruence for transgender and gender-nonconforming (TGNC) individuals, but remains limited by cost and scarcity of specialized providers. Mobile health (mHealth) is a bridge to these access gaps; yet, few high-quality, clinically informed mHealth apps exist within voice clinical sciences.

**Objective:**

We previously created a free mobile app (Attuned; Weill Cornell Medicine and Ithaca College) to deliver clinically informed GAVT through structured instructional videos narrated by a speech-language pathologist and a laryngologist, practice exercises, and pitch-tracking tools, developed with a community advisory board of TGNC individuals. This mixed methods study aimed to assess the app’s usability and users’ impressions.

**Methods:**

Adult TGNC individuals were recruited nationwide via word of mouth and advertising through lesbian, gay, bisexual, transgender, queer, or questioning (LGBTQ+) community organizations. Participants completed a baseline semistructured interview and demographics survey, used Attuned for one week, and completed a second interview regarding their impressions. Interviews were transcribed verbatim and coded inductively in MaxQDA (VERBI Software GmbH). Three authors independently generated codebooks that were reconciled via consensus, then 2 authors applied the finalized codebooks to the remaining transcripts (preconsensus agreement 95.7%; Cohen κ=0.86). Attuned was also evaluated by 2 reviewers experienced in mHealth evaluation and not involved in the app development, using the Mobile App Rating Scale (MARS), the Intercontinental Medical Statistics (IMS) Institute for Health Care Informatics Functionality Scores, the Centers for Disease Control (CDC) Modified Clear Communication Index, the Institute of Medicine (IOM) strategies for creating health-literate mHealth apps, the Patient Education Materials Assessment Tool (PEMAT), and the System Usability Scale (SUS). Readability of the didactic scripts was also assessed with 6 validated formulas.

**Results:**

Eighteen TGNC individuals completed the study (median age 27.5 years, age range 19‐50 years). Ten identified as women and/or transgender women, 6 as nonbinary or gender-nonconforming, and 2 as men or transgender men. Eight themes were identified. Participants identified voice as a crucial determinant of others’ perceptions of their gender, with voice-related dysphoria common. Cost and difficulty finding providers were primary barriers to GAVT, and existing free resources were viewed as unstructured and untrustworthy. Postuse impressions were largely positive, particularly regarding the structured curriculum, pitch tracker, convenience, and free access, though lack of individualized feedback was a key limitation. Mean scores were 4.21/5 (SD 0.53) for MARS; IMS functionality, 6.5/11 (SD 0.7); CDC index, 82.5% (SD 24.7%); IOM strategies, 28.5/33 (SD 2.1); and PEMAT understandability and actionability 100%, and SUS 92.5/100 (SD 10.6), with most readability formulas at or below an eighth-grade level.

**Conclusions:**

Voice is central to gender identity for TGNC individuals; yet, structured and affordable GAVT remains largely inaccessible. Attuned was positively received by this initial cohort and scored highly on usability, health literacy, and readability, though findings are limited by potential acquiescence bias and the raters’ authorship roles.

## Introduction

Transgender and gender-nonconforming (TGNC) individuals are people whose gender identity is distinct from the sex they were assigned at birth [1]. Prior studies have reported that as many as 96% of TGNC individuals report experiencing voice-gender incongruence, defined as a lack of alignment between an individual’s gender and the perceived gender of their voice [2]. Voice has been shown to be intricately linked to TGNC individuals’ self-perception of gender identity, with subsequent profound effects on quality of life [3,4]. Voice modification is thus a part of many individuals’ transition; the standard of care for these patients, potentially alongside hormonal and/or surgical treatments, is gender-affirming voice training (GAVT). GAVT has a growing evidence base supporting its efficacy, both for objective voice parameters and for patient-rated satisfaction [5-10]. While many individuals focus on pitch modification for gender affirmation, many other factors, including resonance, word choice, syntax, and intonation, influence perception of vocal femininity or masculinity, solidifying the crucial importance of GAVT for optimal gender-affirming voice modification [11-13]. However, there is a relative scarcity of qualitative research on GAVT. This is a significant shortcoming given the known difficulties of quantitative measurements in this area, including poor correlation between various patient-reported outcome measures and objective acoustic measurements [5,14].

Although 70% of TGNC individuals desire gender-affirming voice care, they face barriers to health care at much higher rates than the general population [15-18]. There is thus a significant unmet need for GAVT among TGNC patients, fueled by many factors such as relative scarcity of qualified and affirming providers, difficulty obtaining insurance coverage or acquiring necessary funds to pay out-of-pocket, and the fears of many TGNC individuals in engaging with the health care system [16,19,20]. This gap also has implications for research into the demand and desires for GAVT, since both quantitative and qualitative studies are primarily limited to TGNC individuals who are already presenting for GAVT, thus missing the broad swath of individuals who are not able to access it.

Mobile health (mHealth) represents a promising avenue to reduce such gaps in access for this important care, but uptake of mHealth in voice-focused health care by laryngologists and speech-language pathologists (SLPs) remains limited [21]. A recent publication by our group identified fewer than 30 clinically relevant apps within laryngology, many of which scored poorly on evaluations of usability, health literacy, and readability [21]. The relative paucity of mHealth in our field is particularly striking given that conditions under the purview of laryngology—such as chronic cough, gastroesophageal reflux disease, dysphagia, and dysphonia—are extremely common. As the capabilities of digital health and mHealth tools grow, there remains a critical unmet need for high-quality, evidence-based, and accessible mHealth apps within laryngology.

Our group has previously described the creation of a free mobile app providing clinically informed GAVT, “Attuned” (Weill Cornell Medicine and Ithaca College) alongside a community advisory board (CAB) of TGNC individuals, with the goal of closing the gap in delivery of crucial gender-affirming health care [22]. Attuned consists of multiple modules of instructional videos covering voice physiology and laryngeal anatomy, pitch, intonation, resonance, volume, articulation, and syntax. Rather than offering separate feminization and masculinization tracks, each module presents the relevant vocal parameter as a spectrum and teaches techniques for shifting the voice in either a more feminine or more masculine direction, allowing users, including nonbinary users whose goals may not align with a binary framework, to apply the same curriculum toward their individual voice goals. The curriculum is narrated by an SLP and a laryngologist who deliver gender-affirming care in their clinical practice; this content is based on the standard of care in GAVT and was iterated with feedback from the CAB to provide maximum understandability and utility to the target user base. The app also includes other features, such as assigning “homework” assignments and practice exercises, the ability to record voice samples and track vocal pitch over time, and a piano keyboard to provide reference pitches for pitch modification.

In this study, we undertook a mixed method approach to evaluate Attuned for the first time: (1) a qualitative study investigating TGNC individuals’ preexisting perceptions of their voice and voice modification as well as their reactions and feedback following the use of Attuned, and (2) a battery of validated scales to assess the app’s usability, functionality, and readability.

## Methods

### Overview

We used a convergent mixed methods design [23], in which qualitative data (semistructured interviews) and quantitative data (validated mHealth assessment scales and readability measures) were collected in parallel and analyzed independently and integrated at the interpretation level through narrative weaving by comparing qualitative themes with quantitative scale domains.

### Qualitative Study

To maximize diverse subjective feedback, a qualitative study design was used to capture TGNC individuals’ baseline attitudes about voice and gender and their reactions to the Attuned app. The qualitative component of this study is reported in accordance with the COREQ (Consolidated Criteria for Reporting Qualitative Research) [24]; the completed checklist is provided in Checklist 1. Adult (≥18 years) individuals identifying as transgender, nonbinary, or gender-nonconforming were recruited via word of mouth and advertising through lesbian, gay, bisexual, transgender, queer, or questioning (LGBTQ+) community organizations nationwide. Participants were required to have access to an iPhone, since the first version of Attuned was available only for iOS at the time of our study. Recruitment was particularly focused on representing a wide array of gender identities, and sample size was determined by thematic saturation [25,26]; in total, 18 individuals participated.

Following informed consent, participants engaged in a one-on-one semistructured interview with one of 2 members of the research team (ILA or CPK) and completed a demographic survey. They were then given access to the Attuned app for one week and instructed to experience as much didactic material as possible. Following one week, participants were re-engaged for an additional semistructured interview, focused on their perspectives of the Attuned app and its delivery of GAVT. Screenshots of the app are shown in Figure 1. Interview questions were devised by the study team to collect information regarding (1) baseline attitudes about the voice and its relationship with gender, (2) prior experience with and impressions of voice training or self-driven voice modification, (3) impressions of the Attuned app in particular, and (4) reflections on the value and shortcomings of GAVT delivered by mHealth. Interviews were conducted via Zoom (Zoom Communications, Inc), and recordings were transcribed verbatim using a combination of Zoom’s built-in transcription (using a Health Insurance Portability and Accountability Act [HIPAA]–compliant Zoom platform) and the Whisper transcription app by OpenAI (which was run locally and through terminal, thus also achieving HIPAA compliance) [27]. Transcriptions were reviewed for accuracy by the individual who conducted the interview. All interviews took place between February and April 2025.

Interview transcriptions were coded using MaxQDA software (VERBI GmbH). Coding followed an inductive thematic analysis as described by Braun and Clarke [28]. Three authors (ILA, CPK, and RC) independently reviewed and coded the transcripts of the first 3 participants, both pre- and post-use interviews (ie, 6 transcripts in total). The 3 codebooks were then compared and reconciled through consensus discussion among all 3 coders to generate a finalized codebook. Prior to consensus discussion, inter-rater reliability of coding was calculated within MaxQDA, and the percent agreement was 95.7%, with a Cohen κ of 0.86, corresponding to near-perfect agreement. Two authors (ILA and CPK) then independently applied the finalized codebook to all remaining transcripts, with coding differences again reconciled via consensus discussion. No code required adjudication by a third coder. New codes arising during coding of the remaining transcripts were added to the codebook by consensus, and previously coded transcripts were reviewed again with the updated codebook. Thematic saturation was assessed following the approach of Guest et al [25], defined a priori as the point at which successive interviews yielded no new codes or themes. Codes were grouped into candidate themes and consolidated into 28 subthemes, which were reviewed and refined by the coding team into the 8 final themes reported below.

**Figure 1.**
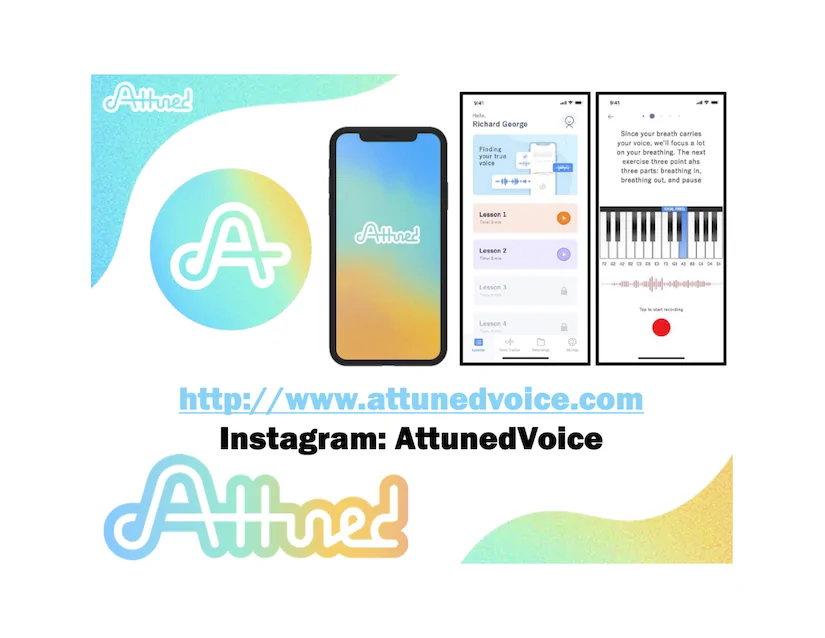
Logo and screenshots of Attuned app interface.

### Objective Assessment of Attuned

Two authors (IC and EO), with previous experience evaluating mHealth apps via validated scales and who were not involved in the development of Attuned, completed ratings via Google Forms. In line with prior systematic assessments of mHealth apps by members of our authorship team [21], the following scales were used: (1) the Mobile App Rating Scale (MARS) [29], (2) the Intercontinental Medical Statistics (IMS) Institute for Health Care Informatics Functionality Scores [30], (3) the Centers for Disease Control (CDC) Modified Clear Communication Index [31], (4) the Institute of Medicine (IOM) strategies for creating health-literate mHealth apps [32], (5) the Patient Education Materials Assessment Tool (PEMAT) [33], and (6) the System Usability Scale (SUS) [34]. No single validated scale captures quality, functionality, health literacy, and usability, so we applied the same multiscale battery used in our group’s prior systematic assessment of laryngology apps [21] to permit direct comparison with other apps. Scores were averaged between the 2 raters. For each of these scales with explicitly numerical output evaluated by 2 raters, interrater reliability was evaluated via calculation of an intraclass correlation coefficient (CA-ICC). Readability of the script used for didactic videos was also assessed using 5 validated readability formulas via Readable.io (Added Bytes Ltd) [35]: (1) Flesch-Kincaid Reading Level and Flesch-Kincaid Reading Ease [36], (2) the Gunning Fog Index [37], (3) Coleman-Liau Index [38], (4) Simple Measure of Gobbledygook (SMOG) Index [39], and (5) New Dale-Chall [40]. As previously described, it is standard to use multiple readability scales, as there is no established gold standard [41]. Each readability formula is calculated differently, focusing on various attributes of a given text selection such as word and sentence length, number of unfamiliar words, number of syllables, and sentence structure complexity. Briefly, the Flesch-Kincaid Reading Ease score ranges from 0 to 100 and is inversely related to readability, while the remaining scales describe a grade level of education necessary to understand the text [41].

### Positionality Statement

The research team would like to formally acknowledge that our individual and collective identities may influence our research. We have opted to share information about the identities of the primary members of the research team who designed and conducted the majority of the present study.

ILA and CPK are both medical students and cisgender members of the LGBTQ+ community and are both advocates for gender-affirming care and the TGNC community. AR is a fellowship-trained laryngologist at the Sean Parker Institute for the Voice at the Weill Cornell Medical College, who completed her training at Stanford University and then at the University of California, Davis. She is a cisgender member of the LGBTQ+ community and an ally of the TGNC community. KAC is a fellowship-trained laryngologist at Stony Brook Medicine specializing in LGBTQ+ care, who trained at Oregon Health & Science University and then at the Sean Parker Institute for the Voice. He is a cisgender member of the LGBTQ+ community and an ally of TGNC people. MP is an SLP who trained at Ithaca College and later returned to direct the speech-language pathology clinic at the same institution. She is a cisgender woman and an LGBTQ+ ally. RC is a voice-specialized SLP, focusing on care of the professional voice and gender-affirming voice care (among other clinical specializations) at the Sean Parker Institute for the Voice, which trained at the Massachusetts General Hospital’s Institute of Health Professions. She is a cisgender woman and an ally to the LGBTQ+ community. All 6 team members underwent cultural humility training prior to beginning the development process.

### Ethical Considerations

This study received approval from the Weill Cornell Medical College Institutional Review Board (#22‐09025226). Informed consent was waived for this study, as the research presented no more than minimal risk to the participants and involved no procedures for which written consent is normally required outside of the context of research. Participants were provided with a study information sheet prior to joining the study. Participants were assigned a participant ID in REDCap (Vanderbilt University), and all survey data were captured in REDCap. Interviews were conducted over Zoom, and once the interviews were transcribed and all personal identifiers were redacted from the transcript using Whisper by OpenAI, which was run offline, the audio recordings of the interviews were destroyed upon completion of the study. Audio recordings and deidentified transcripts were stored locally on a password-protected tagged device and labeled with the participant ID. Participants received US $30 for their participation in the study and were compensated by ClinCard (Greenphire LLC).

## Results

### Qualitative Study

#### Participant Characteristics

A total of 18 interviews were conducted from February to April 2025. Of the 18 participants, 10 identified as women and/or transgender women, 6 identified as nonbinary, gender-nonconforming, or gender fluid, and 2 identified as men or transgender men. Ten used the pronouns she/her, 2 used the pronouns he/him, 4 used the pronouns they/them, one used the pronouns she/they, and one used the pronouns he/she/they. Ten individuals were assigned female at birth (AFAB), 7 were assigned male at birth (AMAB), and one was intersex.

Twelve participants had a history of hormone replacement therapy. Of the 12 participants, 3 had received testosterone treatment and 9 had received one or more of the following treatments: estrogen, progesterone, and spironolactone. Twelve participants also self-reported as being under the neurodivergent umbrella.

The median participant age was 27.5 years, with ages at the time of interview ranging from 19 to 50 years. A summary of these and additional participant characteristics, including geographic regions and settings (urban, suburban, or rural) as defined by the US Census Bureau, is shown in Multimedia Appendix 1.

#### Overall Qualitative Results and Themes

Prior to consensus discussion, interrater reliability was calculated within MaxQDA (VERBI Software GmbH) software; percent agreement was found to be high at 95.7%, with a Cohen κ of 0.86, corresponding to near-perfect agreement. Eight overarching themes were identified (5 from pre-Attuned interviews and 3 from post-Attuned interviews) and will be described individually below: (1) the voice is essential to gender identity, (2) dissatisfaction with the voice is the norm, (3) high-quality voice training is largely inaccessible, (4) navigating and modifying the voice across social contexts, (5) current apps fall short of user needs, (6) Attuned provides a clear curriculum with useful features on users’ own schedules, (7) Attuned can positively impact the voice, and (8) Attuned is limited by lack of immediate feedback and personalization.

#### The Voice Is Essential to Gender Identity

Exemplar quotes pertaining to the importance of voice to gender identity are shown in Table 1. Many participants emphasized that their voice was a key part of their own self-conception of their gender and gender presentation as well as their self-confidence (Q1-Q3), while also stressing its “paramount” effects on others’ perceptions of their gender (Q4-Q7). The voice was identified as a “tell” or a “giveaway” when they felt they could otherwise “pass” or be identified as a cisgender person of the gender to which they had transitioned [42]. Many participants felt that their voice was a reason for being misgendered or assigned a gender different from the one with which they identified (Q8-Q11) [43]. Importantly, several participants noted that being either misgendered or identified as a transgender person may have implications for their safety.

**Table 1.** Exemplar quotations: the voice is essential to gender identity.

Subtheme	Exemplar quotations
Voice’s impact on self-conception of gender	Q1: “It’s how I express myself. All the little sounds I make, everything that I say, it’s just a part of who I am. It’s important.” (P1) Q2: “I think having the right pitch can really help me in my transition and formation of my gender identity. It is really important in the transition process and becoming blended into society as the person and gender I identify with.” (P2) Q3: “What’s most important to me is how I see myself, and just having confidence in my voice, which would make me speak up more and use my voice.” (P16)
Voice’s impact on others’ perceptions of gender	Q4: “I think my voice is very important because the first time I open my mouth when I’m talking to someone, they can easily assume my gender based on my voice and how I talk.” (P2) Q5: “If you know the old saying: ‘If it walks like a duck, looks like a duck, and quacks like a duck, then it’s a duck.’ Well, I look like a duck, but I don’t quack like a duck. So, yeah, it’s one of those things where I’ve seen people turn around expecting me to be a guy because they hear my voice, and they turn around and are confused because I’m not a guy.” (P16) Q6: “I’d say it is paramount to passing in my everyday life.” (P21) Q7: “The fact that it’s possible that I could be passing without it and then completely tarnish it the second I speak is something that weighs on me every single day.” (P28)
Misgendering and safety	Q8: “If people can't see me physically like on the phone or at the drive through, they say ma’am. They assume based on the way they hear my voice that I’m a woman.” (P5) Q9: “You know, there are situations where either for professionalism or for safety or just comfort, it is helpful or convenient to have voice modification available.” (P11) Q10: “But when I had shorter hair and more loosely fitting clothing, my gender became a question and you could tell because people start giving you the look and are wondering to themselves ‘What's going on? What do I say?’ But then, I would open my mouth and they would immediately gender me as a woman.” (P12) Q11: “Yes, I have been misgendered on the telephone. Certainly, in face-to-face situations, where I feel like I am either presenting in a nonbinary, feminine-leaning way. Or, I feel like I'm totally presenting as female, maybe not passing. So it's hard to tell if it's transphobic responses or ignorant, internal bias responses, or just responding to my voice, but I feel like my voice ‘clocks’ me a lot and gets me misgendered.” (P35)

#### Dissatisfaction With the Voice Is the Norm

Exemplar quotes regarding participants’ current dissatisfaction with their voice are given in Table 2. Many individuals cited particular aspects of their voice that they were dissatisfied with, including pitch being too high or too low, or a lack of control over specific sounds, while others felt that their voice was generally “not the way I would like my voice to be” (Q12-Q15). In addition, voice was identified as a source of dysphoria, defined as stress derived from incongruence between one’s gender identity and the gender they were assigned at birth [44]. Some participants noted their voice was a “main cause” for their dysphoria (Q16-Q18). Dysphoria related to the voice was often severe enough to make participants “cry about it” or require them to “pause and...reset at times in conversation.” Accordingly, there was a substantial desire to modify the voice, ranging from technical changes such as pitch modification to more abstract feelings of wanting to “pass” or to align their voice with “how I feel inside” (Q19-Q23). Several participants also shared their experiences with voice modification via gender-affirming hormones, particularly testosterone for voice masculinization. Some individuals feared the potentially irreversible effects of hormones, while others who had used testosterone noted that despite improvements in vocal pitch, the hormone did not allow them to achieve a voice truly in line with cisgender men, but rather often outed them as transgender (Q24-Q30).

**Table 2.** Exemplar quotations: dissatisfaction with the voice is the norm.

Subtheme	Exemplar quotations
Dissatisfaction with voice	Q12: “I am not satisfied with much because it’s not really the way I would like my voice to be.” (P2) Q13: “When I try to just let myself be heard, it tends to just go much deeper than I intended to and I don’t realize until I’m mid-sentence.” (P13) Q14: “I’d say that the satisfaction with my voice has gone down over time, as the rest of me has evolved to fit my ideal image that then it just makes the voice even more important, because it’s kind of like the last piece.” (P16) Q15: “I’m dissatisfied with the fact that it gives me away and the fact that it is masculine.” (P28)
Voice causes dysphoria	Q16: “It’s the main cause of my dysphoria.” (P21) Q17: “It depends on the day. It depends on how I’m feeling. I’d say my voice definitely causes moderate dysphoria. But there are some times where it’s mild and I forget about it. There are sometimes where it’s severe and I cry about it.” (P28) Q18: “Recently I’ve been experiencing quite a bit of dysphoria at times surrounding my voice, to the point where I sort of pause, and have to close my eyes and reset at times in conversation.” (P35)
Desire for voice modification	Q19: “Just being able to talk to somebody in the bathroom would be really great without having anyone feel uncomfortable.” (P1) Q20: “I want my voice to be deeper.” (P6) Q21: “I would like to present in the world based on how I feel inside.” (P10) Q22: “I guess my goal would be probably to 100% pass as a female.” (P16) Q23: “I would just like to be able to speak in a softer, more breezy, feminine way, and do so naturally. It’s a lot of effort at the moment to even try to think about it, and I feel silly at times, even practicing.” (P35)
Impact or perceptions of gender-affirming hormones	Q24: “It feels almost like a catch 22, like, yes, I want my voice to be deeper and all of these things. I want to do this masculinizing therapy. But people’s perception of my voice being different and my vocal cords changing with the testosterone too, causing my voice to be nasally, would then actually make me feel probably more dysphoric than whatever voice my voice had been before.” (P6) Q25: “My voice was changing, and people could tell that it was changing. So their perception of my voice was like, oh, that person is trans or queer. So that was suddenly a reason that I had to start coming out, because my voice was obviously changing and things about my body were changing.” (P6) Q26: “HRT is really interesting and but not something that feels right for me at this time and it feels too permanent.” (P17) Q27: “I definitely still have the voice crack, the pubescent boy voice crack, which is not what I want to sound like.” (P19) Q28: “One of the things I’ve noticed in the past year on HRT is not only does my voice sound different, but I get like vocal cord exhaustion in a way that I never did before. So I talk a lot and I get more tired from it, which is a new experience.” (P19) Q29: “There’s a particular sound to a trans guy kind of voice, and I feel like people hear that particular kind of quality and they notice that it’s different enough and funny-sounding enough that it triggers a pause and curiosity response. And I think that is a significant part.” (P19) Q30: “But overall, in terms of just pitch in general, just talking, it does feel better.” (P19)

#### High-Quality Voice Training Is Largely Inaccessible

Exemplar quotes pertaining to experiences and perceptions of formal or informal voice training are given in Table 3. A few participants recounted mixed impressions of formal voice training with a one-on-one coach or SLP; while such endeavors were often productive, they often failed to meet initial expectations, potentially yielding frustration after spending “so much money on therapy” and still being “misgendered over and over and over again” (Q45-Q46). Some participants also highlighted the utility of nongender-focused voice training for better voice control, which could be leveraged in service of gender-oriented voice modification (Q47). However, the majority of participants had not had prior experience with formal voice training of any kind, citing several key barriers to accessing it, including cost (“prohibitively expensive”), lack of specialized providers, lack of convenience or difficulty with time commitment, and difficulty navigating the landscape of resources to find a voice coach (Q48-Q52). Participants also shared various impressions of free, publicly available resources such as YouTube (Google LLC) videos or posts on social media forums, including Reddit (Reddit, Inc) and TikTok (ByteDance). While such resources provided some benefit, particularly when created by fellow members of the transgender community, navigating the unstructured information from countless sources was often frustrating, and the information shared was sometimes either not what participants were looking for or perceived as unreliable (Q53-Q57). Thus, many individuals still felt a strong desire for formal voice training in order to have a professional guide them through the steps and different aspects of voice modification (Q58-Q61).

**Table 3.** Exemplar quotations: high-quality voice training is largely inaccessible.

Subtheme	Exemplar quotations
Prior experience with voice training	Q45: “I had a couple sessions for vocal training but that was really hard to sit in front of somebody on Zoom and try to change your voice.” (P1) Q46: “I had a moment a few months ago of feeling like I spent so much money on therapy and I spent so much time and effort on this and I was just getting misgendered over and over and over again. I had a period of time where it just kept happening and it was getting really frustrating. Sometimes it bothers me. Sometimes it doesn’t. And I’ve had panic attacks over it. But it’s not a consistent thing.” (P11) Q47: “I am lucky in that I have vocal training as an actor and so I feel like I have more control of my voice than I may have otherwise.” (P17)
Difficulty accessing formal voice training	Q48: “I am working 2 jobs so I cannot make a weekly hour session with either a speech therapist or vocal trainer.” (P12) Q49: “I have considered doing vocal training. But then, you know, it’s obviously not covered by insurance and that’s something that I’m paying for out of pocket. And because of that, it does not become a priority.” (P12) Q50: “Then, you have to find one for a really specific thing. You might even get someone who is not the right fit. Being nonbinary, it’s a little more fuzzy because I am not looking for someone to tell me, ‘Here’s how you speak like a man’ because I am not into that.” (P12) Q51: “I’m aware that there are like people who do voice coaching specifically for people in my situation. I’ve looked into it, but it’s prohibitively expensive.” (P19) Q52: “I’m not even sure where to find one. Like vocal coach dot com? I think I just don’t know where or who or how to acquire one.” (P31)
Experience with publicly available resources	Q53: “I never had consistency with those people. Like I see a TikTok video and they say some things and I can try them on my own, but I don’t have anything consistent.” (P6) Q54: “I would like to have resources to have a lower pitch feminine voice, but all the resources I have access to are attempting to teach you to do use a hyper-femme voice before saying that you can maybe drop it down later. But there’s no explicit path on how to do that.” (P21) Q55: “The voice resources I’ve tried so far, I would give them probably like a 5 or 6 out of 10. They really haven’t yielded much success, but they haven’t hurt either.” (P21) Q56: “I have accessed resources in an attempt to start doing something many times. I just it’s very hard for me without like any sort of structure or guidance from interacting with another human or anything like that. I need something to set the routine for me.” (P28) Q57: “I have used a lot of YouTube videos. And I have found web page apps that that will help track certain aspects of my voice, and let me know what I have to work on. Although it’s always hard to know what to trust on those apps, because some of them are not very accurate.” (P36)
Desire for voice training	Q58: “I think if I get good training and I can change my voice, it can help, as long as it is also coupled with my physical appearance and clothing and such things. It can help people identify my gender much faster than before.” (P2) Q59: “It would probably be really effective to have like a one-on-one voice coach who was like talking me through doing things.” (P3) Q60: “I think a voice coach would probably be one of those things that would make a humongous difference. There’s the technical aspects of your voice, but there’s also ways of explaining things or saying things that are considered more masculine or feminine-like. So, someone that could have an outside perspective on how I’m being read or perceived would be something I would want.” (P16) Q61: “With any sort of training, control is really nice, and when you have control and knowledge, I think it gives you a lot of autonomy.” (P17)

#### Navigating and Modifying the Voice Across Social Contexts

Exemplar quotes regarding self-guided attempts at voice modification are shown in Multimedia Appendix 2. Many participants, largely without any formal instruction, focused primarily on attempts to alter their pitch to better align with their gender (Q31-Q33). Some also identified other aspects of voice that they sought to modify for gender-affirming purposes, such as cadence, resonance, weight, and volume (Q34-Q36). For most participants, such modifications were situational, changing their voice in professional situations or based on the individuals to whom they were speaking, such as using a “girl voice…with my friends…then at work…I use my guy voice” (Q37-Q42). Some also highlighted a fear of vocal injury or a feeling that altering their voice felt “forced” or “uncomfortable” (Q43-Q44), especially in the absence of reliable instruction for voice modification.

#### Current Apps Fall Short of User Needs

Exemplar quotes for participants’ prior experience with voice-focused apps and their priorities for an ideal voice-focused app are shown in Multimedia Appendix 3. Several participants reported using apps largely focused on pitch identification and classification of the voice as male, androgynous, or female, though many had doubts about the accuracy and reliability of such apps (Q62-Q65). Participants were also asked to identify key priorities for their ideal voice-focused app. Common priorities included personalization for an individual’s particular goals, a clean user interface, the ability to play back recordings or receive automated feedback, clear information and instructions, and structured teaching with the ability to practice (Q66-Q71).

#### Attuned Provides Clear Curriculum With Useful Features on Users’ Own Schedules

The final 3 sections describe themes from the interviews conducted following the week during which participants used the Attuned app. Exemplar quotes regarding participants’ overall satisfaction with Attuned and their perceptions of its particular strengths and benefits can be found in Table 4. All participants expressed overall positive impressions of the app, and several stated they “would definitely recommend” it to others (Q72-Q76). Two areas of particular strength were the clear structure and order with which participants were guided through the curriculum (Q77-Q80)—a significant improvement over attempts to cobble together other “scattered and unorganized” free resources—and the pitch-tracking function, which is coupled with a piano keyboard for pitch identification and pitch targeting (Q81-Q83). Participants also highlighted the convenience of app-based delivery of GAVT, allowing people to learn at their own pace and use the app in a variety of settings (Q84-Q87). Access to this free app also eliminated price as a major “sticking point” for many individuals. Finally, participants appreciated the clear delivery of instruction and emphasized the feeling of trustworthiness that comes from receiving information from a trained speech-language pathologist and a team of creators who “know what they’re doing” (Q88-Q91).

**Table 4.** Exemplar quotations: Attuned provides clear curriculum with useful features, on users’ own schedules.

Subtheme	Exemplar quotations
Overall satisfaction with Attuned	Q72: “It’s much better than any other voice app that I’ve used.” (P5) Q73: “I’m not aware of any robust voice training services available like this. And I think it’s very beneficial. I would definitely recommend for anybody who was trying to have some at home practice, either in between speech therapy sessions or by itself.” (P11) Q74: “I do think getting information about your body is very empowering, and I think that is something it does very well. I think it’s information a lot of people don’t have and would benefit from.” (P19) Q75: “I think it’s a really wonderful starting point terms of educating you on terms and the different qualities of the voice that make it up. It’s amazing.” (P28) Q76: “I think that if you don’t have access to an in-person voice therapist, I think the app is a lot better than having to pore through forums and YouTube videos and stuff like that.” (P32)
Appreciation of clear structure	Q77: “I thought the general structure of progressively getting more difficult and having a pretty clear line of progression was useful for me.” (P3) Q78: “It’s difficult, if you’re just trying to do voice training in a DIY context or on your own, because everything is so scattered and unorganized in terms of community resources. So that aspect of it was very helpful of just saying, OK, do this and this and this and this and then do these things together.” (P11) Q79: “And throughout it, I really enjoyed how it built on each aspect. Especially if somebody’s coming in without much knowledge of what goes into what makes a voice work and language and all of that, it can be really informative.” (P17) Q80: “I really liked the structure. It’s so important in terms of like educating you on the terms and finding the motivation. It’s the only reason I had never started before just because of the overwhelming nature with zero guidance. This puts you on a certain path and it’s nice.” (P28)
Pitch tracking function was particularly helpful	Q81: “I thought that the little keyboard tool to show what frequency your voice is currently at and where you want to go was really great and helpful for getting a better sense of where I want to be.” (P10) Q82: “I don’t tend to think of voice in terms of like ‘Oh, these are actual like pitches, and these notes, have names.’ So that was very interesting to actually be able to match and see that visually.” (P12) Q83: “I liked the piano. That was really helpful. And I liked the option to record right there. Some of the exercises I wouldn’t have been able to do if I couldn’t both be playing the note and also trying to make a noise.” (P19)
Convenience and accessibility were key benefits	Q84: “Today I was doing it during my dog walk, or last night I was working on it while I was lying in bed. The other day I did a couple of minutes, when I had nothing to do at work. So I like that you can do little spurts.” (P6) Q85: “I could be listening to things right before I go into a meeting or spaces where I know that people are going to be meeting me for the first time. And I want to have that moment of listening to the articulation lesson or returning to a practice, whenever it is most needed.” (P17) Q86: “I think access is the biggest benefit, because that is such a sticking point for so many people. Like, even if their insurance covers it, the logistics of that are a bear.” (P32) Q87: “For many of us who identify as transgender, it is nice that it is private. I felt safe and secure doing the training in the comfort of my home.” (P36)
Information was clearly delivered and felt reliable	Q88: “It was obviously designed in close partnership with people who very much know what they’re doing. So that definitely comes through in a way that makes it both fundamentally useful, but also credible.” (P12) Q89: “I think it does a really good job of entry-level, accessible education around what one can control around their voice.” (P17) Q90: “It is very legitimizing to have it come from an actual speech therapist.” (P32) Q91: “The material felt digestible and easy to understand. I also found that the length of each lesson was appropriate.” (P36)

#### Attuned Can Positively Impact the Voice

Exemplar quotations focused on Attuned’s effects on the voice are given in Multimedia Appendix 4. Some participants felt that the app had already begun to affect changes in their voices, both in terms of their own satisfaction with their voice and the way their voice was perceived by others (Q92-Q94). However, the majority of participants had not yet noticed changes in their voice after only a week but remained optimistic that the tools and skills they had learned from the app would impact their voice with time and dedicated practice (Q95-Q98).

#### Attuned Is Limited by Lack of Immediate Feedback and Personalization

Exemplar quotations pertaining to challenges and drawbacks of using Attuned, or app-based delivery of GAVT in general, are shown in Table 5. A primary concern shared by many participants was the lack of feedback from the app, with several expressing uncertainty whether they were performing the exercises correctly; the loss of face-to-face communication, including potential physical touch from a coach, was also highlighted (Q99-Q101). Relatedly, some participants also felt that the app had a limited ability to personalize the curriculum based on their particular goals and felt that a “personal relationship” with a voice coach was also a meaningful aspect of voice training that was lost via an app (Q102-Q104). Since the app was in its initial beta-testing phase during this study, many individuals also cited specific technical glitches, which sometimes made the app difficult to navigate or operate (Q105-Q107). Finally, some participants requested that more voices, particularly trans voices or voices representing different dialects of English, be included in the instructional content, since only cisgender voices lead to participants “not hearing [themselves]” represented (Q108-Q110).

**Table 5.** Exemplar quotations: Attuned is limited by lack of immediate feedback and personalization.

Sub-Theme	Exemplar quotations
Difficult to not receive feedback from another person	Q99: “People are really key for how one speaks. You can be really loud and resonant on your own, but if you feel nervous in front of other people, that’s something that can only come with practice. Working with a coach, or even small groups of other people doing it would help break down that closing in on your throat when you feel nervous. So I think most apps run into the need for human connection at some point.” (P17) Q100: “There’s no feedback of like, ‘Yes, that is actually the note you’re making.’ I have no idea. So there’s a guessing element to it. That’s an element of ‘I don’t really know if this is right.’” (P19) Q101: “I think the lack of feedback from someone who’s actually hearing you is something that makes a difference. So, if you have the means, I would still recommend going to actual voice training over just using an app.” (P32)
Lack of personalization and personal relationship	Q102: “It’s not necessarily tailored to what you would want, instead of like getting that one on one work with like a speech pathologist or someone.” (P10) Q103: “I think that obviously you have a less personal relationship. It’s an app versus actually building a client provider relationship. So there is a bit of that disconnect.” (P12) Q104: “I’d say that it’s a very useful tool, specifically for people who are beginning their voice training journey, because what it lacks in personalization and makes up for with general ease of use.” (P21)
Beta-testing version of app had glitches	Q105: “The app was glitchy for me. Stuff didn’t load consistently.” (P3) Q106: “There were a couple of technical things about the app that I figure were because it’s in its beta stage. Sometimes I would have to close the app and reopen it to get things to work.” (P6) Q107: “I will say that they did have some technical difficulties with using the app.” (P10)
Diversity of voices in video content would be appreciated	Q108: “Obviously, I think there is some level of comfort that could be achieved, even an aspirational goal that you could assign by receiving training from somebody who is themselves a trans person who has pursued and completed voice training.” (P11) Q109: “I feel like there could have been more of a diversity of the trainers, which I figure you guys also know with the app very much in testing stages. I know that there were a few things read by the more masculine person, or maybe just one. But having a more diverse set of voices would be great. And it was all in standard American. So having like AAVE^a^ or other dialects would be cool and great to see.” (P17) Q110: “I would have really appreciated if there were some trans voices. Cis women’s voices, I know what those are supposed to sound like. And there was an element of not hearing myself that I think we’re all very used to.” (P19)

a AAVE: African-American Vernacular English.

### Objective Assessment

MARS, SUS, and IMS scales were used to evaluate quality and functionality. The overall mean MARS score averaged between the 2 raters was 4.21/5 (SD 0.53), with the following scores for each subcategory: engagement 4.40/5 (SD 0.57), functionality 4.13/5 (SD 0.18), aesthetics 4.17/5 (SD 0.71), information 4.33/5 (SD 0.47), and subjective quality 4.0/5 (SD 0.71). However, interrater reliability, as measured by CA-ICC, was poor (0.348). The mean SUS score was 92.5/100 (SD 10.6), with an excellent CA-ICC of 0.912. The IMS score was 6.5 (SD 0.7) of the 11 recommended functions. Functions that both reviewers found to be missing were the ability to share and evaluate data, the provision of guidance based on user-entered information, and the provision of communication between health professionals and patients.

Next, Attuned was assessed with regard to health literacy via the CDC, IOM, and PEMAT scales. The CDC Clear Communication Index was 82.5% (SD 24.7 percentage points), slightly below the recommended “passing” score of 90%; missing elements largely pertained to the use of language, which is likely to be used by the audience, and to the use of chunked or bulleted text. The CA-ICC for this measure was also poor (0.347). The mean IOM score was 28.5 (SD 2.1) of 33 recommended strategies implemented within Attuned, with the “engage users” and “evaluate and revise your site” domains scoring lower. Both PEMAT actionability and understandability scores were 100%, with complete agreement between reviewers with a CA-ICC of 1.00.

Readability as determined by the Flesch-Kincaid Reading Level and the Gunning Fog Index scores was 5.6 and 7.9, respectively, both below the recommended eighth-grade reading level. The Flesch Reading Ease was 72.3, corresponding to roughly a seventh-grade reading level. However, the SMOG index was 9.3, signifying that 9.3 years of school education would be necessary to read the text, and the Coleman-Liau index also resulted in an eighth-grade reading level at 8.7. On the other hand, the New Dale-Chall score was 4.5, roughly corresponding to a fourth-grade reading level.

### Integration of Qualitative and Quantitative Findings

Qualitative and quantitative findings were integrated through narrative comparison and demonstrated substantial convergence. The high SUS score (92.5/100) converged with participants’ qualitative descriptions of the app as clear, well-structured, and easy to navigate (Theme 6). The IMS functionality assessment identified missing functions, such as provision of guidance based on user-entered information and communication between users and professionals, that mirrored the qualitative limitation theme of absent feedback and personalization (Theme 8). Similarly, the CDC Clear Communication Index fell below the passing threshold primarily on use of the audience’s own language, converging with participants’ requests for trans voices and greater dialect diversity in instructional content. Divergence was minimal: the strong PEMAT and readability results were consistent with participants’ descriptions of content as “digestible and easy to understand.”

## Discussion

### Principal Findings

In this mixed methods study, we have used qualitative methodology to (1) describe baseline attitudes toward voice and vocal modification among transgender individuals and (2) evaluate impressions of a free mobile app delivering clinically informed GAVT. We have also used a wide variety of validated mHealth-focused scales to assess the mobile app for functionality, usability, health literacy, and readability. Given the increasing focus on gender-affirming health care in the United States, the importance of both research on gender affirmation and easily accessible health interventions for TGNC individuals cannot be overstated. The findings of this study help to further establish the widespread demand and need for GAVT and to understand diverse user experiences with an initial iteration of an open-access novel GAVT mobile app for planned further improvement.

GAVT alone or in combination with surgery has been repeatedly shown to improve objective measures such as fundamental frequency, listener perceptions, and patient-reported outcome measures [45-47]. However, the vast majority of existing evidence is for voice feminization, since exogenous testosterone lowers vocal pitch among transmasculine individuals taking gender-affirming hormones, though many such individuals still report voice problems or dissatisfaction with their voices [48,49]. In addition, compared to the growing quantitative literature, relatively few qualitative studies have been undertaken to deepen understanding of factors affecting patients that are not adequately reflected in quantitative measures.

Our study uncovered several key themes among our study population at baseline, prior to use of the Attuned app. Participants consistently emphasized the importance of their voices as a key facet of their complete gender identity and as a determinant of their ability to be perceived by others in line with their gender. This is in line with prior qualitative studies of both transfeminine and transmasculine individuals, which have identified both internal self-confidence and experiences of misgendering as intimately linked to the voice [50-52]. An additional qualitative study by Holmberg et al [53] also noted the key contributions of the voice toward dysphoria, similar to our findings; although they did not explicitly explore dissatisfaction with the voice in parallel, they also identified a strong motivation among their participants to seek voice modification. Importantly, our inclusion of transgender men and nonbinary individuals alongside transgender women in our study population allowed for key perspectives regarding both trepidation and relative dissatisfaction with certain aspects of testosterone’s effects on the voice, highlighting that testosterone alone is sometimes insufficient for achieving vocal congruence. This solidifies the importance of further study among individuals seeking voice masculinization and further use of GAVT among this population. The phenomenon of an outwardly evident “trans guy kind of voice” that can present barriers to “passing” for transgender men is in line with existing knowledge that gender perception goes beyond simple pitch changes. A small number of previous qualitative studies have also raised similar concerns among transgender men who remain dissatisfied or continue to experience misgendering even after long-term testosterone use [52,54]. Furthermore, several nonbinary participants explicitly refrained from a binary feminization/masculinization framing of voice goals, describing both voice coaching and existing apps as engaging in “gender-boxing” that rendered them unusable (Q55, Q74). This underscores that voice modification goals cannot be inferred from gender identity alone and that both GAVT services and mHealth tools should accommodate individualized, nonbinary voice targets.

Contrary to most existing voice-related qualitative research among TGNC individuals, our study population consisted largely of individuals who desired but did not feel that they had access to GAVT. Our cohort also included individuals across a wide range of gender identities, geographic locations, ages, and prior experiences with various gender-affirming treatments. This allowed for substantial exploration of several areas that are not robustly represented in existing literature. For example, our findings regarding self-guided attempts at voice modification, including some practices that participants themselves worried could be causing vocal injury, offer an important glimpse into the vocal practices of TGNC individuals in a landscape of formal GAVT being inaccessible to many. Our findings regarding the importance of context in determining voice use and attempts at voice modification were in line with prior research [52]. Furthermore, our study uncovered not only themes regarding the inaccessibility of formal voice training but also several patterns of frustration with both publicly available online materials and prior voice-centered mobile apps, which substantially builds on prior work by Bush et al [55]. This represents a shortcoming of prior qualitative research in this field, which has largely recruited individuals already presenting to SLPs for GAVT, thus failing to capture the broad swath of the TGNC population who do not reach this point due to previously mentioned substantial barriers.

Our results also represent a largely positive reception among the first cohort of target users of Attuned from diverse geography, age groups, and gender identities. These results should be interpreted in light of the risk of acquiescence bias, given the interviewers’ membership in the development team, and are best understood as encouraging formative feedback rather than evidence of effectiveness. Despite technical glitches in the initial version of the app, participants looked favorably on the app’s content, structure, and tools to support their voice modification efforts. Participants emphasized the empowering nature of receiving detailed information about the mechanics of the voice and gender perception, in line with prior qualitative studies of individuals who have undergone GAVT [56,57]. Of note, qualitative literature investigating outcomes of GAVT is remarkably sparse, despite known shortcomings of quantitative measurements in this realm, such as lack of correlation between different patient-reported outcome measures or between these measures and objective acoustic outcomes [5,14]. This suggests that existing literature may not be adequately capturing the full depth of TGNC individuals’ experiences with GAVT and identifies a crucial area for further investigation.

Our study also uncovered substantial shortcomings of an app-based delivery of voice training, most prominently the lack of feedback and personalization. While certain aspects of these limitations will be intrinsic to any mobile app, advances in AI and biofeedback may allow future versions of Attuned to respond more robustly in real-time to users [58]. An additional consideration for app-based GAVT is the absence of clinical screening prior to training. In many institutional settings, laryngoscopic examination is recommended before initiating voice training to exclude underlying laryngeal pathology, whereas mobile apps are by nature accessible without professional evaluation. Attuned addresses this in part through an in-app disclaimer encouraging users with voice symptoms to seek evaluation by an otolaryngologist. Moreover, our qualitative findings suggest that in the current landscape, many TGNC individuals without access to formal care already attempt self-guided voice modification using unstructured online resources (including practices participants themselves worried could cause vocal injury). Structured, professionally designed guidance with appropriate safety recommendations may therefore represent a safer alternative to the unstructured resources many users currently rely on. It is also important to note that while some mobile apps focused on GAVT exist, most existing offerings range from free pitch-tracking with limited instructional material to structured instructional content available by subscription only [58-63]. TruVox (University of Cincinnati), a free web-based application, provides real-time pitch and resonance feedback alongside instructional content [58]. To the authors’ knowledge, Attuned is distinctive in combining a free sequential video curriculum narrated by voice clinicians with practice assignments and pitch-tracking in a native mobile app developed with a CAB.

With regard to the objective assessment of Attuned, scores on the battery of validated scales were high across quality, functionality, health literacy, and readability, though these tests were performed by members of the authorship team and should be interpreted accordingly. Attuned exceeded the previously published means of laryngology-focused mobile apps across all evaluated scales, notably achieving above 4/5 on the MARS scale and all associated subcategories, 100% on the PEMAT actionability and understandability scales, and resulting in scores below the benchmark of an eighth-grade reading level for the majority of readability scales [21]. Of note, our prior systematic assessment of laryngology mobile apps [21] did not use the SUS; however, Attuned’s score of 92.5 was well above the previously reported benchmarks of 68 and 80 across all digital health tools [64]. This reflects the goal of our app design and content creation process to develop a tool that would be readily available to users from around the country and with varying educational and socioeconomic backgrounds [22]. In addition, it should be noted that the version of the app subject to evaluation on these scales was the first version of the app ever made available and contained known glitches and deficits, which have already been targeted for improvement in future iterations. Future iterations of Attuned addressing these known deficits may perform differently, which will require further evaluation.

Despite its novel contributions, this study is not without limitation. First, while TGNC community members were intimately involved in the creation of Attuned and early plans for the qualitative portion of this study, we did not follow a strict community-based participatory research methodology without a governance structure, authorship opportunities, and fair compensation as previously described [22]. Given our group’s aspirations toward true community-based participatory research [22], this represents a crucial area of improvement for future studies of Attuned. A new CAB is currently being assembled to guide the revision process of the app, as well as to participate as co-investigators in future prospective research using Attuned.

While our use of semistructured interviews allowed deeper exploration of important themes, the necessary trade-off is that interviews were slightly different from each other and could not be directly compared. In addition, participants may have been subject to acquiescence bias, particularly in the post-Attuned interviews, whereby individuals may have felt subtle pressure to overemphasize positive attitudes toward Attuned, knowing that the interviewers were part of the app’s development team. We sought to partially mitigate this bias by asking open-ended questions to signal openness to any opinions that participants would share. Additionally, the objective assessment scales were completed by 2 authors. Although they were not involved in the app’s development, they were not independent of the study team, and these ratings therefore cannot be regarded as fully unbiased. Usability measures were not collected directly from study participants, which we have incorporated into the design of our planned prospective follow-up study. Furthermore, we did not apply exclusion criteria to potential participants (aside from requiring that they were identified within the TGNC umbrella). Our study may therefore be limited by selection bias, as participants choosing to participate in a study of GAVT are likely already interested in GAVT and are also highly motivated to pursue it; our study population—despite our best efforts to recruit broadly from across the country and to include a variety of gender identities and socioeconomic statuses—may thus have limited generalizability to the population of TGNC individuals at large. Participants’ specific voice modification goals (feminization, masculinization, androgynous, or other targets) were not captured as a survey item. Although gender identity distribution offers an approximation, these do not map neatly onto one another, and explicit characterization of voice goals will be incorporated into future studies. Our study did not collect backend information regarding each participant’s usage pattern or time spent on the app, which may have allowed for deeper analysis of participants’ impressions of the app. Finally, both interviewers are cisgender men and members of the LGBTQ+ community but not the TGNC community; this positionality, as previously mentioned, may have impacted the responses of some participants, who may have responded differently to non-LGBTQ+ interviewers or fellow TGNC individuals.

Future directions for our team include 2 directions. First, a revision of the Attuned app based on our participants’ feedback is currently underway, guided by a newly assembled CAB and addressing the technical issues identified in this study and expanding the diversity of voices represented in the instructional content. Planned future capabilities include personalized AI-driven guidance and real-time voice biofeedback to address the feedback and personalization limitations identified by participants, with development contingent on available funding and technical resources. Our team also plans a prospective, quantitative assessment of the app’s efficacy, including participant-administered usability measures, as compared with standard-of-care in-person GAVT. The present study also highlighted several important future directions for the field, including prioritization of studies of transgender men or other individuals seeking voice masculinization, outreach to populations without access to traditional GAVT, and expansion of overall qualitative study of TGNC individuals either seeking or after voice modification training in order to identify shortcomings and further improve outcomes for these individuals.

### Conclusion

The voice is a crucial part of identity and interpersonal interactions for TGNC individuals, and many desire tools for voice modification but have difficulty accessing high-quality GAVT. In a landscape full of unstructured free resources of varying levels of trustworthiness, there is a critical unmet need for a structured, clinically informed curriculum that can be accessible at scale. Attuned, a free mobile app developed through a user-centered approach, was positively received by an initial cohort of TGNC users. While app-based training cannot replace the individualized feedback of one-on-one voice training, these findings suggest promise in expanding access to structured, professionally developed GAVT, to be confirmed in a prospective comparative study.

## Supplementary material

10.2196/96102 Multimedia Appendix 1 Demographic data for participants in qualitative study.

10.2196/96102 Multimedia Appendix 2 Exemplar quotations: navigating and modifying the voice across social contexts

10.2196/96102 Multimedia Appendix 3 Exemplar quotations: current apps fall short of user needs.

10.2196/96102 Multimedia Appendix 4 Exemplar quotations: Attuned can positively impact the voice

10.2196/96102 Checklist 1 COREQ checklist.

## Abbreviations

AFAB: assigned female at birth
AMAB: assigned male at birth
CA-ICC: calculation of an intraclass correlation
CAB: community advisory board
CDC: Centers for Disease Control
COREQ: Consolidated Criteria for Reporting Qualitative Research
GAVT: gender-affirming voice training
HIPAA: Health Insurance Portability and Accountability Act
IMS: Intercontinental Medical Statistics
IOM: Institute of Medicine
LGBTQ+: lesbian, gay, bisexual, transgender, queer, or questioning
MARS: Mobile App Rating Scale
mHealth: mobile health
PEMAT: Patient Education Materials Assessment Tool
SLP: speech-language pathologist
SMOG: Simple Measure of Gobbledygook
SUS: System Usability Scale
TGNC: transgender and gender-nonconforming
