# Supplementary material for: Gender-Affirming Voice Training Delivered by a Novel Mobile App: Mixed Methods Study
Source: JMIR Mhealth Uhealth. 2026 Oct 8;14:e96102. doi: 10.2196/96102 (PMC13649169; doi:10.2196/96102)
Supplement: Supplementary file 2 [file mhealth-v14-e96102-s001.docx]

**Supplemental Table 1.** Demographic data for participants in qualitative study.

| **Participants, N = 18** | **N (%)** |
| --- | --- |
| **Sex Assigned at Birth**  Assigned Female at Birth (AFAB)  Assigned Male at Birth (AMAB)  Intersex | 10 (55.6)  5 (27.8)  1 (5.5) |
| **Gender Identity**  Woman or transgender woman  Non-binary, gender non-conforming, or gender fluid  Man or transgender man | 10 (55.6)  6 (33.3)  2 (11.1) |
| **Pronouns Used**  She/her  He/him  They/them  She/they  He/she/they | 10 (55.6)  2 (11.1)  4 (22.2)  1 (5.5)  1 (5.5) |
| **History of Gender-Affirming Hormone Therapy**  Estrogen, progesterone, and/or spironolactone  Testosterone  None | 9 (50.0)  3 (16.7)  6 (33.3) |
| **Age (years)**  <21  21-25  26-30  >30 | 2 (11.1)  4 (22.2)  6 (33.3)  6 (33.3) |
| **Race**  White  Black or African American  Other | 14 (77.8)  2 (11.1)  2 (11.1) |
| **Employment Status**  Employed  Unemployed  Not in labor force | 15 (83.3)  2 (11.1)  1 (5.5) |
| **Level of Education**  Some college, no degree  Bachelor’s degree  Graduate or professional degree | 6 (33.3)  6 (33.3)  6 (33.3) |
| **Geographic Area**  Northeast  Midwest  South | 9 (50.0)  3 (16.7)  6 (33.3) |
| **Geographic Setting**  Urban  Suburban  Rural | 14 (77.8)  1 (5.5)  3 (16.7) |
