# Supplementary material for: Gender-Affirming Voice Training Delivered by a Novel Mobile App: Mixed Methods Study
Source: JMIR Mhealth Uhealth. 2026 Oct 8;14:e96102. doi: 10.2196/96102 (PMC13649169; doi:10.2196/96102)
Supplement: Supplementary file 3 [file mhealth-v14-e96102-s002.docx]

**Supplemental Table 2. Exemplar Quotations: Navigating and Modifying the Voice Across Social Contexts**

| **Sub-Theme** | **Exemplar Quotations** |
| --- | --- |
| Self-guided pitch alteration | Q31: “I found this one video that showed me how to elevate your Adam's apple to have higher pitch, and I did that. It did not come out as squeaky, but it came out as like definitely a dude trying to sound like a girl, and I knew it just did not sound right.” (P1)  Q32: “I’m just trying to not go into that highest register, because that just always sounded not right to me.” (P19)  Q33: “I think I've just tried to more consistently talk in either a lighter or a higher register, just in a way that seems generally more perceivable as feminine.” (P32) |
| Attempts at modification of other vocal elements | Q34: “I usually think of it like speaking softer. I guess I alter moreso the cadence and the tempo of speaking, and not so much trying to alter my voice, but alter my speech.” (P16)  Q35: “I try to speak higher, softer, more windy, and I use more of an up and down kind of way of talking and try to end my sentences on a higher pitch to try to attempt to sound more feminine.” (P35)  Q36: “I also raise the resonance, and I lower the vocal weight.” (P36) |
| Altering voice in particular situations | Q37: “If it's with my grandparents, then it is a bit more monotone and a little bit deeper. At work, it's a little bit more elevated, but not more feminine. And then in social situations, it's just bubbly and feminine.” (P1)  Q38: “When I'm with new people or people who don't know me well, I try to change my voice a little bit.” (P2)  Q39: “I try to explore going into the deeper range, especially if I'm in a queer space.” (P6)  Q40: “I do a lot of customer service and interaction with the public as a part of my work. And so one of the main things I do for my full time job is answer the phone. And it is really weird to see how people really try to seek gendering things over the phone. And so for most of them, the only part of me they know is my voice. Definitely working in that environment, I find myself pitching up.” (P12)  Q41: “I'll fall back into the habit, especially when I'm supposed to be like the authoritative source on something, and I'll kind of bring out more of the guy voice.” (P16)  Q42: “I have two really distinct voices. I have my girl voice, which I use when I'm with my friends, I'm chilling out, life is good. Then at work because I work in construction, I use my guy voice. It's great. It's fine. It's just such a jarring difference. Like if someone hears me over the phone versus seeing me at work, it's like a completely different person almost.” (P31) |
| Fear of vocal injury or experience of “forcing” voice | Q43: “I used to try to force a voice all the time and it was so uncomfortable and it just made me feel worse.” (P1)  Q44: “I try not to think about pitch too much because honestly I don't think I can do much to go a lot higher. I just kind of aim for more of an androgynous range when it comes to pitch because I don't want to hurt myself.” (P11) |
