# Supplementary material for: Gender-Affirming Voice Training Delivered by a Novel Mobile App: Mixed Methods Study
Source: JMIR Mhealth Uhealth. 2026 Oct 8;14:e96102. doi: 10.2196/96102 (PMC13649169; doi:10.2196/96102)
Supplement: Supplementary file 4 [file mhealth-v14-e96102-s003.docx]

**Supplemental Table 3. Examplar Quotations: Current Apps Fall Short of User Needs**

| **Sub-Theme** | **Exemplar Quotations** |
| --- | --- |
| Prior voice app use | Q62: “I'm sure it was lovingly designed. But, you know, I wondered if it is accurately picking up my vocal tones.” (P12)  Q63: “It was one of those pitch detector apps. And it was annoying because it would tell you if your pitch was in a feminine, masculine, or androgynous range. Even my partner, who was a cis woman at the time was in the masculine range. It felt like a sketchy sort of algorithm thing that did not work well.” (P32)  Q64: “I did use one for a day, and then I just stopped using it. I deleted it or something, because it wasn't very good.” (P32)  Q65: “There's an app that I've used a couple of times, just to kind of see where I am and how comfortable it is to get my voice into the neutral area. The app works by your voice being blue, or male, neutral for non-binary, and then pink for female. So yeah, I have tried to see how comfortable it is for me to talk in those female pitches.” (P35) |
| Priorities in an ideal voice app | Q66: “I think also the knowledge and remembrance that, yes, people are probably going for either vocal feminization, or vocal masculinization. But I would want the app to not necessarily have just those two boxes. Because for someone like myself who is non-binary, some of the apps do fall into this gender-boxing and then it makes the app not useable for myself. So, remembering that people's goals with the app might be very specific and very individual, and not fit into a larger preset category.” (P12)  Q67: “Something that I have noticed with language learning apps like Duolingo, is their user interface feels cluttered. So I would want something that is just very concise with a clean user interface.” (P13)  Q68: “I want it to be more than just instructions on the screen. Like, if it could give you feedback and say, "Hey, you're doing a good job." Or, have the app listen to you and be able to give you feedback without you having to go and try out your voice on real people.” (P16)  Q69: “Also, I think that breaking down concepts into simple, easy to understand modifications to a voice and explaining the interplay between them and constructing a voice that you want is really important.” (P21)  Q70: “I think that audio examples and exercises and also potentially a way of creating a practice regimen, or something like that, seem like the most useful things.” (P32)  Q71: “Having a playback feature, so that when I've done well, or when I've done poorly, I can listen to my own voice, and try to hear what those differences are.” (P36) |
