# Supplementary material for: Gender-Affirming Voice Training Delivered by a Novel Mobile App: Mixed Methods Study
Source: JMIR Mhealth Uhealth. 2026 Oct 8;14:e96102. doi: 10.2196/96102 (PMC13649169; doi:10.2196/96102)
Supplement: Supplementary file 5 [file mhealth-v14-e96102-s004.docx]

**Supplemental Table 4. Exemplar Quotations: Attuned™ Can Positively Impact the Voice**

| **Sub-Theme** | **Exemplar Quotations** |
| --- | --- |
| Perception that voice has already changed somewhat | Q92: “The app impacted my satisfaction with my voice. Particularly, it helped me try to find the right pitch and volume for me. And I feel like it made some positive impact.” (P2)  Q93: “I was working on some exercises last night and then my partner called me on the phone and was like, oh!” (P6)  Q94: “I started a clinical rotation this past week, and I haven't had any instances of being misgendered during that time or any questions suggesting that I was being misgendered, so I think it has impacted others' perception of my voice.” (P32) |
| Optimism that further practice will improve voice | Q95: “It’s not something that you can be like, okay, I'm gonna start speaking 3 registers lower...It takes time. But it takes a bit of the mystery out of it. I now feel like this is something that I could maybe do. Here are the actual steps.” (P12)  Q96: “I feel like it gave me the information I need to know what work I need to do. Here's a bunch of information and if you choose to use it in an ongoing way, then you might feel like you're improving.” (P19)  Q97: “It was a really short amount of time to have that big of an impact, but I do think that I can see the scope for it having a positive impact with more use, for sure.” (P32)  Q98: “I definitely found it helpful. However, I would have to spend more than one week with it to see a difference in my voice.” (P35) |
